# Antimicrobial activity of spherical silver nanoparticles prepared using a biocompatible macromolecular capping agent: evidence for induction of a greatly prolonged bacterial lag phase

**DOI:** 10.1186/1477-3155-8-34

**Published:** 2010-12-21

**Authors:** Peter Irwin, Justin Martin, Ly-Huong Nguyen, Yiping He, Andrew Gehring, Chin-Yi Chen

**Affiliations:** 1 Molecular Characterization of Foodborne Pathogens Research Unit, Eastern Regional Research Center, Agricultural Research Service, U. S. Department of Agriculture, 600 East Mermaid Lane, Wyndmoor, PA 19038 USA; 2 PPG Industries, Pittsburgh, PA USA

## Abstract

**Background:**

We have evaluated the antimicrobial properties of Ag-based nanoparticles (*Np*s) using two solid phase bioassays and found that 10-20 μL of 0.3-3 μM keratin-stabilized *Np*s (depending on the starting bacterial concentration = *C*_I_) completely inhibited the growth of an equivalent volume of *ca*. 10^3 ^to 10^4 ^colony forming units per mL (CFU mL^-1^) *Staphylococcus aureus*, *Salmonella *Typhimurium, or *Escherichia coli *O157:H7 on solid surfaces. Even after one week at 37°C on solid media, no growth was observed. At lower *Np *concentrations (= [*Np*]s), visible colonies were observed but they eventually ceased growing.

**Results:**

To further study the physiology of this growth inhibition, we repeated these experiments in liquid phase by observing microbial growth via optical density at 590 nm (OD) at 37°C in the presence of a [*Np*] = 0 to 10^-6 ^M. To extract various growth parameters we fit all OD[t] data to a common sigmoidal function which provides measures of the beginning and final OD values, a first-order rate constant (*k*), as well as the time to calculated 1/2-maximal OD (*t*_m_) which is a function of *C*_I_, *k*, as well as the microbiological lag time (*T*).

Performing such experiments using a 96-well microtitre plate reader, we found that growth *always *occurred in solution but *t*_m _varied between 7 (controls; *C*_I _= 8 × 10^3 ^CFU mL^-1^) and > 20 hrs using either the citrate-([*Np*] ~ 3 × 10^-7 ^M) or keratin-based ([*Np*] ~ 10^-6 ^M) *Np*s and observed that {∂*t*_m_/∂ [*Np*]}_citrate _~ 5 × 10^7 ^and {∂*t*_m_/∂ [*Np*]}_keratin _~ 10^7 ^hr·L mol^-1^. We also found that there was little effect of *Np*s on *S. aureus *growth rates which varied only between *k *= 1.0 and 1.2 hr^-1 ^(1.1 ± 0.075 hr^-1^). To test the idea that the *Np*s were changing the initial concentration (*C*_I_) of bacteria (*i.e*., cell death), we performed probabilistic calculations assuming that the perturbations in *t*_m _were due to *C*_I _alone. We found that such large perturbations in *t*_m _could only come about at a *C*_I _where the probability of any growth at all was small. This result indicates that much of the *Np*-induced change in *t*_m _was due to a greatly increased *T *(*e.g*., from *ca*. 1 to 15-20 hrs). For the solid phase assays we hypothesize that the bacteria eventually became non-culturable since they were inhibited from undergoing further cell division (*T *> many days).

**Conclusion:**

We propose that the difference between the solid and liquid system relates to the obvious difference in the exposure, or residence, time of the *Np*s with respect to the bacterial cell membrane inasmuch as when small, *Np*-inhibited colonies were selected and streaked on fresh (*i.e*., no *Np*s present) media, growth proceeded normally: *e.g*., a small, growth-inhibited colony resulted in a plateful of typical *S. aureus *colonies when streaked on fresh, solid media.

## Background

In his famous and often cited talk given to the American Physical Society in 1959, Richard Feynman challenged scientists across all disciplines to consider the possibilities that could be achieved by miniaturization and atomic level control. In the ensuing fifty years, significant progress has been made to this end, affording scientists the ability to reproducibly create nanometer-sized inorganic structures including: spheres [1,2], wires [3], rods [3], tubes [3], belts [3], prisms [4-8], dendrimers [9], and many others [10]. As the chemical and physical properties of a nanomaterial are intimately linked to its size and shape, significant effort is, and has, been placed toward the syntheses of novel nanomaterials [11]. The ability to modify physical and chemical properties such as light scattering, absorption and emission, magnetic properties, electrical properties and others toward a specific application have made inorganic nanomaterials suitable for a wide variety of applications. Traditionally, these applications have included sensors, catalysis, electronics, surface enhanced Raman spectroscopy, biology and diagnostic imaging [1,12-14].

Recently, there has been a great deal of interest surrounding the discovery that silver nanoparticles (*Np*s) are significantly more effective antimicrobial agents in terms of the minimum effective concentration than their Ag^+ ^counterparts [15]. This enhancement in relative antimicrobial activity has led researchers to develop their use in conjunction with medical products [16], their fixation on textiles [17-20] and other materials to prevent microbial growth or infections. Thus, one of the greatest challenges in integrating silver *Np*s with commercial products is attaining proper adhesion and functionality throughout the lifetime of the treated product. Unfortunately, we have found that the adhesion of the well-characterized citrate-stabilized silver *Np*s to textiles to be poor. Furthermore, many of the options available for functionalizing the surfaces of textiles such as chemical treatments or cold plasma treatments degrade the material or affect some of their desirable intrinsic properties. To overcome such *Np *limitations, we have been exploring the use of biocompatible protein stabilizers such as keratin to allow for facile attachment of the nanomaterial to textile surfaces through gentle heat or enzymatic processes. This process produced discrete spherical silver *Np*s with a diameter of 3.4 ± 0.74 nm that could be freeze-dried and easily re-suspended in water without ultrasonication and without significant aggregation. As this size distribution is in agreement with that obtained by Mirkin *et al. *[6] in their well-known synthesis of citrate-stabilized *Np*s (4.2 ± 0.9 nm), an opportunity was presented to study the effect of the keratin capping agent and the process of freeze drying/re-suspension on the silver *Np*'s ability to act as an antimicrobial agent. To the best of our knowledge, very little is known about the effect of macromolecular stabilizers on antimicrobial properties and microbial growth kinetics when encapsulating silver *Np*s of similar size and shape [21], nor is the effect of processing the particles via freeze drying well-known. The importance of understanding the impact that a *Np *stabilizer has on antimicrobial properties is highlighted in a recent study by Elechiguerra and coworkers [13] where silver *Np*s were prepared using three different protocols. Their results showed that *Np*s < 10 nm selectively bound to a glucagon-like peptide (glp20) to inhibit HIV-1 and noted that there was a difference in efficacy between the three capping technologies. These authorities suggest that the differences in antimicrobial effectiveness between silver *Np*s capped with polyvinylpyrrolidone (PVP), foamy carbon and bovine serum albumin (BSA) may be due to the manner in which the *Np*s interact with the stabilizer. In the case of a foamy carbon matrix, they believe the *Np*s are virtually free, while for PVP and BSA, the *Np*s were believed to be tethered to the protein and encapsulated, resulting in their slightly reduced antimicrobial efficacy. In addition to stabilizer/surface interaction, the actual arrangement of silver atoms on the *Np *surface may be important. In a recent study, Pal *et al. *[22] suggest that specific surfaces may be important for observing efficacy (*e.g*., the 111 surface: where the surface plane intersects the x-, y- and z- axes at the same value).

In this study, we investigate the growth kinetics and inhibition of one Gram-positive (*Staphylococcus aureus*) and two Gram-negative bacteria (*Escherichia coli *O157:H7 and *Salmonella enterica *serogroup 'Typhimurium' = *Salmonella *Typhimurium) in the presence of both citrate-stabilized and keratin-capped *Np*s at various concentrations using a real-time spectrophotometric assay (*i.e*., growth-related behavior in aqueous media). We also investigate the effect of freeze-drying and resuspension on *Escherichia coli *and *Salmonella *Typhimurium. For comparison purposes we performed two solid-state Petri plate-based assays (*i.e*., behavior on solid media).

## Results and Discussion

### Inhibition of *S. aureus *Growth on Solid Media

Table 1 shows spread plate colony count data resulting from an inoculum of 500 μL of *S. aureus *(*i.e*., a 10^-4 ^dilution of an overnight culture = 10^-4 ^× *C*_0 _= *C*_I _~ 6 × 10^4 ^CFU mL^-1^) being dispersed across standard (80 cm^2^) Brain Heart Infusion (BHI) Petri plates. After drying the plates, 10 μL of various concentrations of freeze-dried keratin-capped silver *Np*s were applied drop-wise to the surface using a 6-channel pipette (*i.e*., 6 observations per region) across 4 regions per spread plate. After overnight growth at 37°C, we saw that there were distinctive circular areas (~ 0.3 cm^2^) of limited *S. aureus *growth: *i.e*., at the higher [*Np*]s, what colonies existed were much smaller than those observed growing outside these zones. Upon counting what colonies appeared, we saw that the counts decreased linearly with *Log*_10_[*Np*]. Analysis of variance and a multiple range test were performed (Methods Section); any 2 averages were considered significantly different at the *p *= 0.05 level if the absolute value of their difference was $>\;q_{0.05}\;s_{\bar{x}}$. We also noted that the small colonies within the zone of growth inhibition did not appear to grow further while those outside the inhibition zone of each *Np *drop grew into each other forming an almost contiguous colony. Interestingly, after several days of no apparent growth, when one of these growth-inhibited colonies was sampled and streaked on fresh media (*i.e*., in the absence of silver *Np*s), there was a proliferation of normal colony growth. This result implies that the continued presence of the keratin-capped silver *Np*s on the plate's surface limited further cell division. Table 1 also indicates that a ratio of at least 10^11 ^*Np*:CFU is required to show complete growth inhibition. Similar results were observed for both *Salmonella *Typhimurium and *E. coli *O157:H7 (data not shown).

**Table 1:** Spread plate growth of *Staphylococcus aureus* on solid media in the presence of various *Np* concentrations.

	***CFU cm* ^-2^**					
			
**[*Np*](nM)**	**Region: 1**	**2**	**3**	**4**	**$\bar{x}$ ± *s***	
					
2903	22	30	35	6	23 ± 13	*a*
2322	58	67	75	42	61 ± 14	*b*
1742	118	61	127	82	97 ± 30	*b*
1161	148	116	126	83	118 ± 27	*b*
290	325	234	287	380	307 ± 62	*c*
145	321	327	327	329	326 ± 3	*c*
29	399	355	479	414	412 ± 51	*d*
0	314	349	303	361	332 ± 28	*c*

Averages associated with different letters are significantly different at the *p *= 0.05 level (ANOVA & multiple range test performed on log-transformed data). The size of the spotted *Np *areas was approximately 0.255 cm^2^. The lowest effective concentration provides a *Np*:CFU ratio of ca. 10^11^. This calculation is assuming a [*Np*]-0 CFU intersection occurring at about 2 × 10^13 ^*Np*s (in 10 μL) and 375 CFU per 0.255 cm^2 ^drop area.

In order to improve the experimental variation, we performed a drop plate-based assay (Table 2) that would provide better control for dispensing the test organism on the plate's surface. This protocol involved first placing twenty (4 × 5 format using a 4-channel pipette) evenly spaced 20 μL bacteria-laden drops (~ 10^-5 × ^*C*_0_= *C*_I _= 2 × 10^3 ^CFU mL^-1^; BHI-diluted) onto each of 2 plates. Then, after drying, 4 × 20 μL of each *Np *concentration (up to *ca*. 0.8 μM) was carefully added on top of each air-dried, organism-loaded spot. Growth at 37°C was checked daily for at least a week. Each drop plate set was replicated thrice using different *S. aureus *cultures and dilutions (Methods Section). Multiple range tests were performed on both the *linear *and *Log*-transformed data. In these experiments we saw that no *S. aureus *colonies developed when *ca*. 0.4 to 0.8 μM keratin-capped *Np*s were applied. At lower [*Np*]s colony counts were linearly related ([*Np*] ≥ 0.2 μM) with *Log*_10_[*Np*]. As before (Table 1), the observed colonies that formed were small and appeared to remain *in stasis*, or only grew at a much reduced rate, relative to those colonies forming in the control (*i.e*., [*Np*] = 0) areas or at much lower [*Np*]s.

**Table 2:** Drop plate growth of *Staphylococcus aureus* on solid media in the presence of equivalent volumes (20 μL) of various *Np* concentrations.

	***CFU mL* ^*-1*^**					
				
**[*Np*] (nM)**	**Exp: 1**	**2**	**3**	**$\bar{x}$ ± *s ***	****log****	****Linear****
				
783	0	0	0	0		
587	0	0	0	0		*a*
392	0	0	0	0		*a*
196	88	25	88	67 ± 36	*a*	*a*
157	375	125	288	263 ± 127	*b*	*ab*
117	463	413	438	438 ± 25	*bc*	*ab*
78	763	450	725	646 ± 171	*c*	*b*
39	1638	1050	1813	1500 ± 399	*d*	*c*
20	2350	1388	1913	1883 ± 482	*d*	*cd*
0	2163	1913	2050	2042 ± 125	*d*	*d*

Averages associated with different letters are significantly different at the *p *= 0.05 level (ANOVA & multiple range test performed on both **log**- transformed {null values were excluded} and non-log-transformed or **linear **data). The lowest effective concentration (~ 392 nM) provides a *Np*:CFU ratio of ca. 10^11^.

Interestingly, at the outer boundaries of each *Np *drop there was a continuous ring of *S. aureus *growth which never impinged within the well-defined zones of inhibition. These data indicate that the maximum keratin-based silver *Np *growth inhibition was observed at a *Np*:CFU ratio of about 10^11 ^which is similar to that observed previously (Table 1). Growth-inhibited colonies when streaked on fresh media grew normally, however, after several weeks of no observable growth on the original *Np*-treated regions, spread plating of one of these small colonies on fresh media resulted in no growth. This observation indicates that these cells were either moribund, or, more likely, dead.

### Inhibition of Bacterial Growth in Liquid BHI

Table 1 and 2 clearly demonstrated that on a solid matrix, where both bacteria and *Np*s have limited motion, the keratin-based silver *Np*s completely inhibited *S. aureus *growth. Would a similar effect occur in a liquid where bacteria and *Np*s can both move freely? To answer this question and potentially gain some insight into the physiology involved, OD-based growth assays [23] were performed and a large set of treatments (*e.g*., 11 levels of [*Np*]s {5, 10, 15, 20, 25, 30, 35, 40, 45, and 50 μg per well ≅ 0.26, 0.52, 0.78, 1.0, 1.3, 1.6, 1.8, 2.1, 2.3, and 2.6 μM} + 1 negative control + 3 keratin only controls all in BHI; *C*_I _= 8.3 × 10^3 ^CFU mL^-1 ^± 13%) were distributed in a 96-well microtitre plate. The covered plate was equilibrated at 37°C for a short period of time and OD (λ= 590 nm) measured after shaking every 14 min for over 25 hrs. From the OD[t] truncated data arrays, **Eq. 1 **(all equations are discussed in the Methods section) was used and the various growth parameters (*k *and *t*_m_) were determined.

Analysis of variance was performed on both parameters and we found that there was no statistically significant effect of the various [*Np*]s on *k *(*F*_13,26 _= 3.6; $\;k\pm q_{0.05}s_{\bar{x}}÷2=1.1\pm 0.075\;{\text{hr}}^{-1}$; doubling time = τ = 38 ± 2.6 min). However, there was a significant effect on *t*_m _(Table 3), which is the incubation time to 1/2-maximal OD (OD_F _÷ 2, **Eq. 1**). It is important to keep in mind that by the time we begin to observe an increase in OD, about 10-15 doublings will have occurred. Because of this fact, the OD-based lag time (*t*_m_: **Eq. 1**) [24] is related to the starting cell concentration (*C*_I _), the rate of growth (*k*), as well as the microbiological lag time (*T*) [23]. These interrelationships are fully developed in **Eq. 5**.

**Table 3:** Dependency of *Staphylococcus aureus* t_m_ on keratin-stabilized *Np* (freeze-dried) and associated probabilities (P_+_) that the changes in t_m_ are due to perturbations in the C_I_ (~ 8 × 10^3^ CFU mL^-1^) in the presence of the Nps.

**per well**		***t*_m _(hrs)**						
			
***μ*g *Np***	***μ*g keratin**	**Exp: 1**	**2**	**3**	**avg**		***P*_+, _avg**	***T*_corr, avg_(hrs)**
							
0	0	6.74	6.13	6.89	6.59	*a*	1	1.11
5	0	8.72	7.18	8.52	8.14	*a*	1	2.66
10	0	13.3	11.4	12.3	12.3	*b*	0.798	6.83
15	0	13.4	12.8	13.9	13.4	*b*	0.644	7.91
20	0	15.7	14.7	15.6	15.4	*c*	0.0995	9.89
25	0	16.8	15.7	16.3	16.2	*c*	0.100	10.8
30	0	19.3	18.2	17.8	18.4	*d*	0.00551	13.0
35	0	23.5	20.3	21.4	21.7	*e*	0.00163	16.3
40	0	24.9	21.7	24.8	23.8	*f*	0.0000628	18.3
45	0	27.1	26.9	27.3	27.1	*g*	0.00000288	21.6
50	0	27.6	28.4	26.8	27.6	*g*	0.00000165	22.1
0	10	6.65	6.11	6.89	6.55	*a*	1	1.08
0	25	6.95	6.20	6.69	6.62	*a*	1	1.14
0	50	6.69	6.00	6.73	6.47	*a*	1	1.00

Averages associated with different letters are significantly different at the *p *= 0.05 level. There is no significant effect of the keratin alone on *t*_m_. The 5 μg *Np *level is equivalent to ca. 2.6 × 10^-7 ^M.

Since the apparent effect *Np*s have on *t*_m _could also result from a change in *C*_I _(via cell death), we have also estimated the probability (*P*_+_, **Eq. 6**) for any growth occurring in the 96-well plates assuming only changes in *C*_I _with a *T *fixed at 1 hr. Therefore, in essence, *P*_+ _is the probability that the observed changes in *t*_m _could be due to perturbations in the *C*_I _in the presence of the *Np*s. These data are also presented in Table 3 and demonstrate that a *t*_m _beyond about 7-9 hrs is highly unlikely to be due to changes in initial bacteria concentration. We calculated a corrected *T *(*T*_corr _= *T*-*T*_C_+1) by assuming that the controls (*T*_C _= 1.1, 1.1, 1.1, and 1.0 hrs for 0 + 0, 0 + 10, 0 + 25, 0 + 50 control combinations {*i.e*., μg *Np *+ μg keratin per well}, respectively, Table 3) have a *T *of ~1 hr which is the approximate true microbiological lag time in unperturbed systems (*T *= 1.4 ± 0.49 hr). When a *T*_corr _was estimated, we saw a linear relationship with [*Np*]: ∂*T*_corr_/∂ [*Np*] ~ 8.3 × 10^6 ^L·hr mol^-1 ^[± 3%], *T*_corr, [*Np*] = 0 _~ 1.1 ± 0.47 hr, r^2 ^= 0.99. To the best of our knowledge, there are no known treatments which can cause such a clear, and relatively predictable, perturbation in bacterial lag times. Thus, in solution, the *Np*s can induce a 20 hr increase in the microbiological lag time but eventually all treatments grow to a normal OD_F _level (Methods section). We propose that the same physiological effect is occurring on solid surfaces but, because the *T *values are so long, the bacteria eventually expire or go into deep stasis.

**Figure 1:**
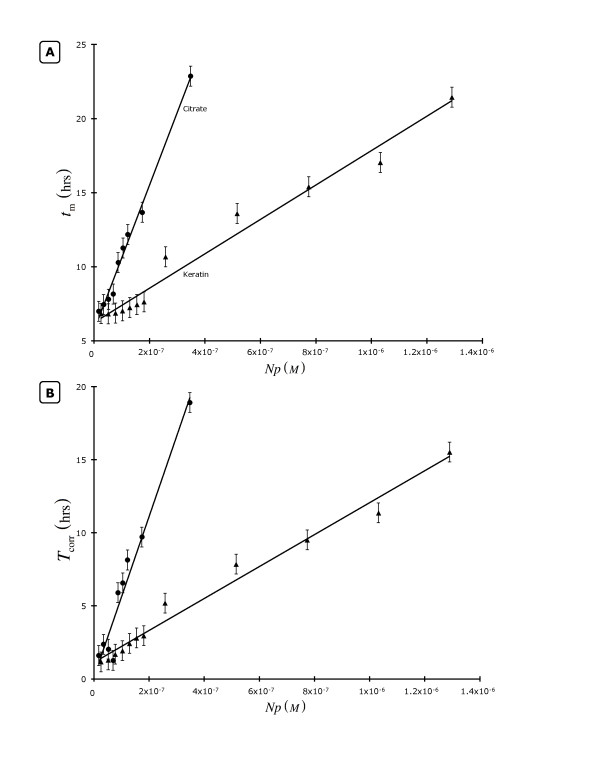
**The dependence of the *Staphylococcus aureus *time to 1/2-maximal OD (*t*_m_; **1A**) and corrected microbiological lag time (*T*_corr_; **1B**) on citrate- (circles) or keratin-capped (triangles) Ag *Np*s**. All data points represent the mean ($\bar{x}$) of 3 replicates.

For comparison purposes, we investigated the relative efficacy of keratin- and citrate-capped silver nanoparticles. Figure 1 displays both *t*_m_- (**1A**) and *T*_corr_-based (**1B**) averages calculated from *S. aureus *(3 cultures = 3 blocks or replicates) microplate growth assays using either citrate- (●) or keratin-capped (▲) *Np*-treated BHI at 37°C. Both *Np *treatments had a linear relationship with respect to their effect on either *t*_m _(citrate: ∂*t*_m_/∂ [*Np*] ~ 4.9 × 10^7 ^L·hr mol^-1 ^[± 4%], r^2 ^= 0.99; keratin: ∂*t*_m_/∂ [*Np*] ~ 1.2 × 10^7 ^L·hr mol^-1 ^[± 5%], r^2 ^= 0.98) or *T*_corr _(citrate: ∂*T*_corr_/∂ [*Np*] ~ 5.5 × 10^7 ^L·hr mol^-1 ^[± 8%], r^2 ^= 0.95; keratin: ∂*T*_corr_/∂ [*Np*] ~ 1.1 × 10^7 ^L·hr mol^-1 ^[± 4%], r^2 ^= 0.98) as a function of [*Np*]. At low [*Np*]s, both citrate- and keratin-stabilized *Np*-treated cultures asymptote to similar values of *t*_m _(*t*_m,[*Np*] = 0 _= 5.7 ± 0.29 and 6.2 ± 0.33 hr for citrate- and keratin-based Ag *Np*s, respectively) or *T*_corr _(*T*_corr,[*Np*] = 0 _= 0.12 ± 0.67 and 1.1 ± 0.26 hr). Differing from the keratin-capped Ag *Np *behavior we saw previously (*i.e*., on semi-solid surfaces: Table 1 and 2), a greater *Np*:CFU ratio was required (> 10^12^), in order to achieve a maximum growth inhibition effect. From the ratios of slopes (either ∂*t*_m_/∂ [*Np*] or ∂*T*_corr_/∂ [*Np*]) we saw that the citrate-stabilized Ag *Np*s were about 4-5-fold more effective than the keratin-based *Np *at an equivalent *C*_I_. This difference illustrates the value of understanding the effect that a *Np *stabilizer has on antimicrobial properties since it is known that different-sized stabilizers can result in different efficiencies [13]. Our results in Figure 1 indicate that a similar stabilizing agent size-based phenomenon may be occurring with the keratin-capped *Np*s. It is also possible that the keratin-stabilized Ag *Np*s have an activity distribution where ca. 20% are as fully active as citrate-based particles while the rest are completely inactive due to excessive imbedding of the crystalline silver *Np *assembly within the capping protein's structure.

### Anomalous *Np *activity differences in fresh BHI

**Figure 2:**
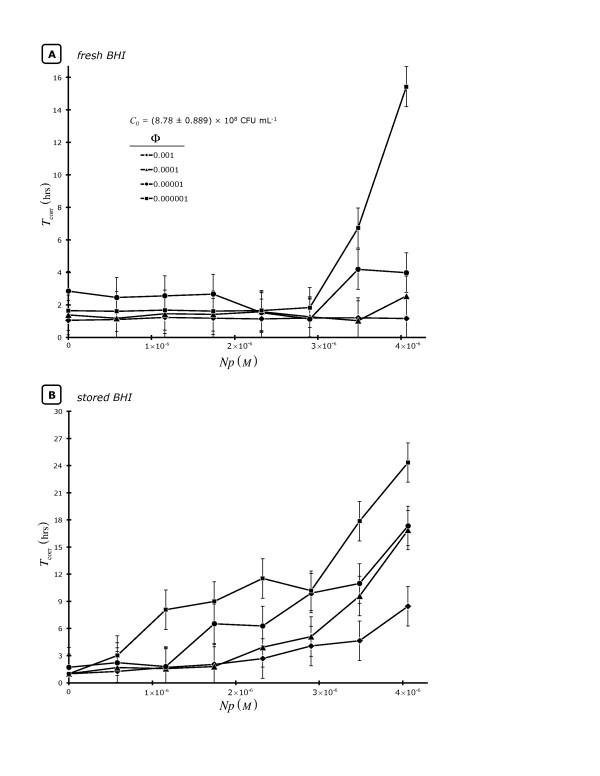
**The dependence of corrected microbiological lag time (*T*_corr_) using fresh BHI (**2A**) or aged BHI (**2B**) on keratin-capped Ag *Np*s at four *Staphylococcus aureus *concentrations whereupon *C*_I _= *C_0_*Φ**. All data points represent the mean ($\bar{x}$) of 3 replicates.

During the course of this study, we noticed an inexplicable change in the response of *S. aureus *to keratin-capped *Np*s, which appeared to be coincidental with a change in liquid media: *i.e*., from that which was stored to that which was freshly made from the same lot of BHI powder. Because of this we performed another set of experiments (Figure 2) to specifically clarify the effects of both media (**2A**: fresh BHI; **2B**: stored BHI) as well as initial *S. aureus *concentration (*C*_I_) on the growth response to keratin-capped *Np*s. Because *C*_I _has such a strong effect on *t*_m _[24], only *T*_corr _averages, calculated from 3 BHI-diluted overnight cultures (*C*_0_) used to generate each initial concentration of *S. aureus*, are reported. To do this, 4 dilutions (the dilution factors, Φ_I_, = 10^-3 ^[◆], 10^-4 ^[▲], 10^-5 ^[●], and 10^-6 ^[■]) from 3 separate *S. aureus *overnight cultures grown in freshly prepared BHI (*C*_0 _= 8.8 × 10^8 ^CFU mL^-1 ^[± 10%]) were created (*C*_I _= *C*_0_Φ_I_), distributed into a 96-well plate and 8 levels of keratin-stabilized [*Np*]s were introduced. Similar to what we have referred to previously (Table 1 and 2, Figure 1), we noted that a large *Np*:CFU ratio (ca. 10^12^; [*Np*] ~ 4 × 10^-6 ^M; *C*_I _~ 8.8 × 10^2 ^CFU mL^-1^) was required to achieve the maximum growth perturbation effect (largest *T*_corr _~ 15 and 24 hrs for fresh or stored BHI, respectively). There were other clear-cut effects of the media aging on *S. aureus*' apparent lag phase response to keratin *Np*s inasmuch as there was almost no significant lag time response to the presence of lower [*Np*] levels relative to the same culture diluted with stored BHI.

**Figure 3:**
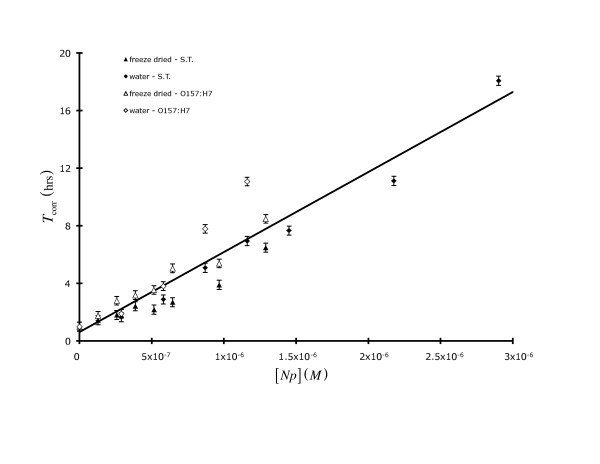
**The dependence of corrected microbiological lag time (*T*_corr_) on either freeze-dried (triangles) or water-based (diamonds) keratin-capped Ag *Np*s for *Salmonella *Typhimurium (solid symbols) or *E. coli *O157:H7 (open symbols)**. All data points represent the mean ($\bar{x}$) of 3 replicates.

Lastly, we sought to determine the relative efficacy of keratin-capped Ag *Np*s (in fresh BHI) with respect to Gram-negative bacilli. Figure 3 shows *T*_corr _data determined from growth studies using a *C*_I _~ 3 × 10^3 ^CFU mL^-1 ^*Salmonella *Typhimurium (closed symbols) or *E. coli *O157:H7 (open symbols), both of which are pathogenic. In these experiments we also characterized these bacteria for their response to *Np*s that were either freeze-dried (triangles) and then re-suspended in fresh BHI or those that were stored in their original aqueous medium (diamonds). As in previous work there was an approximately linear relationship between *T*_corr _and [*Np*] (*e.g*., ∂*T*_corr_/∂ [*Np*] ~ 5.6 × 10^6 ^L· hr mol^-1 ^[± 6%], *T*_corr,[*Np*] = 0 _~ 0.62 ± 0.34 hr, r^2 ^= 0.90). The lag time data presented in Figure 3 indicates that there was not any consistent overall loss of *Np *antimicrobial activity upon freeze drying. Compared to the keratin-based Ag *Np *antimicrobial activity (*i.e*., *Np*:CFU ratio for maximal activity ca. 10^12^) we saw previously with *S. aureus*, the *Np*:CFU ratio which resulted in maximal activity was ca. 10^11^. Thus these particular Gram-negative organisms appear to be more sensitive than *S. aureus *to the keratin-based Ag *Np*s.

## Conclusions

In this work we have evaluated the antimicrobial properties of a biocompatible macromolecular capping agent-based (keratin) Ag *Np *using both solid- and solution-state media assays. We found that on solid surfaces, 10-20 μL of 0.3-3 μM keratin-based *Np*s completely inhibited the growth of *Staphylococcus aureus *and, after several weeks at 37°C, no further growth was observed. At lower *Np *concentrations, intermediate levels of colony formation occurred (less than the control) but the colonies ceased growing beyond a certain small size. When these small colonies were selected and streaked on fresh media without *Np*s, growth proceeded normally. These results imply that further cell division is limited due to the continued presence of Ag *Np*s on the solid surface.

In liquid phase we found that growth always occurred but the *t*_m _varied between 7 and > 20 hrs (assuming a constant *C*_I _) using either the citrate- ([*Np*] ~ 3 × 10^-7 ^M) or keratin-based ([*Np*] ~ 10^-6 ^M) *Np*s. We discovered that this delay was not related to the effect that *Np*s had on *S. aureus k *values. To test the possibility that the *Np*s were effectively changing *C*_I _bacteria via cell death, we performed probabilistic calculations assuming that the perturbations in *t*_m _were due to *C*_I _alone (*i.e*., with a fixed *T*).

We found that our observed large perturbations in *t*_m _could only come about at concentrations where the probability for any growth occurring at all was small. This result indicates that much of the *Np*-induced change in *t*_m _was due to a greatly increased value for the true microbiological lag time (*T *increased from ~ 1 to > 15-20 hrs). In either solution or the solid state, a maximum perturbation was noticed only when the ratio of [*Np*]:*C*_I _(on a particle:cell basis) was about 10^11^-10^12^. We propose that the differences observed between the solid and liquid growth systems relates to obvious differences in the residence time of the *Np*s with respect to the bacterial cell membrane.

## Methods

Scoured and carbonized wool fibers, ~ 21 μm in diameter, were obtained from the Bollman Hat Company, Adamstown PA. Silver nitrate, sodium citrate, sodium borohydride, sodium hydroxide, and methylene chloride were obtained from Sigma-Aldrich and used as received. 6,000-8,000 Da molecular weight cutoff Spectra Por dialysis tubing was obtained from VWR scientific and used as received. Deionized water was obtained using a Barnstead Nanopure filtration system. TEM images were collected using a Phillips CM12 Cryo system. UV-VIS measurements were recorded in solution using a Cary 50 Conc spectrometer, a Tecan Microplate Reader equipped with XFluor4SafireII software v4.62A (100 averages), a Perkin-Elmer HTS7000+ 96 well plate reader (used for bacterial growth data exclusively), and an Aviv instruments UV-VIS spectrophotometer model 14NT-UV-VIS.

### Preparation of keratin hydrolysate

Keratin hydrolysates were prepared by taking cleaned and scoured wool and adding this to a 0.5 N NaOH solution at 60°C for three hours. The hydrolyzed keratin was dialyzed through Spectra Por dialysis tubing with a 6,000-8,000 Da molecular weight cutoff. The water was changed three times during a 24 hour dialysis period. The hydrolyzed keratin was then lyophilized using a FTS Flexidry™System. Upon addition of the protein, a change in the pH toward basic was observed.

### Preparation of colloidal keratin stabilized silver nanoparticles

Stable colloidal Ag *Np*s were prepared by adding 0.1 g of the dried keratin hydrolysate to 100 mL of rapidly stirring deionized water. The pH of the system was adjusted to 8.5-8.9 using a dilute sodium hydroxide solution if necessary. After dissolution, 0.184 g (ca. 10^-3 ^mol) of silver nitrate was added to the stirring keratin solution and the pH was observed to change to approximately 6.7. In a separate vial, 0.0097 g (ca. 2.5 × 10^-3 ^mol) of sodium borohydride was measured and added to 5 mL of deionized water.

Exactly 1 mL of this solution was added dropwise to the rapidly stirring keratin/silver nitrate solution at room temperature over the course of 10 minutes. The solution changed from a clear to dark orange color and the final pH of the solution was measured to be 7.7. The particles were spun in a Cole-Parmer benchtop centrifuge (≤ 3800 RPM) and the liquid fraction was removed with a glass Pasteur pipette. An identical amount of clean deionized water was added and this procedure was repeated at least three times. For lyophilization studies, the silver *Np *suspension was lyophilized using a FTS Flexidry™System.

**Figure 4:**
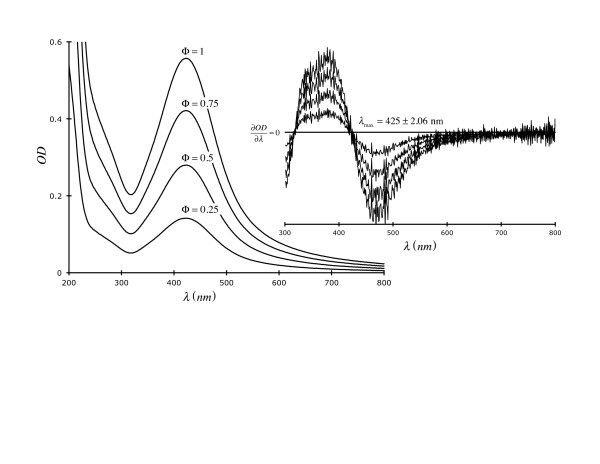
**Absorbance and first derivative spectra of keratin-capped Ag *Np*s at 4 dilutions**. **λ **_max _is an average of the 4 derivatives (at ∂*OD */∂λ = 0).

Figure 4 shows that the maximum OD occurs at λ = 425 ± 2.06 nm (average across 4 dilutions) which is due to surface plasmon resonance, a feature common to sols of discrete inorganic *Np*s. The absorbance at shorter wavelengths is due to π→π* and n→π * transitions from the keratin capping agent. *Np *concentrations were determined spectroscopically according to a previously published procedure [25]. Using TEM, we established that our keratin-based *Np*s are spherical with a diameter (*d *) normally-distributed (unimodal) about *d *= 3.4 ± 0.74 nm (*μ *± σ). Citrate-stabilized Ag *Np*s were prepared and rinsed according to a procedure published by various workers [4-8] .

### Spread plate growth assay procedures

For the spread plate assay 500 *μ*L of a 10^-4 ^dilution (*ca*. 6 × 10^4 ^CFU mL^-1^) of *Stapylococcus aureus *grown in BHI broth overnight at 37°C was evenly spread over the entire surface of a BHI broth-based solid (2% agarose) media Petri plate (*ca*. 80 cm^2^) and allowed to dry 15 min in a microbiological hood to avoid surface contamination. After compete drying, various solutions (from *ca*. 10^-7 ^to 3 × 10^-6 ^M) of the freeze-dried keratin *Np*s which had been suspended in sterile water were applied as 10 μL drops to the plate: 6 drops per region (6 drops each were applied with a multiple channel pipette to the 2 middle and 2 exterior regions of the Petri dish; experiments were replicated this way to take into account the slight variability of spreading the bacterial suspension evenly) and 4 regions per plate in a randomized complete block experimental design where each "region" represents a separate "block". Areas of growth inhibition were measured and colonies were counted several times over the course of a week at 37°C.

### Drop plate growth assay procedures

For the sake of both precision and accuracy, we also performed a drop plate assay which consisted of applying 4 × 5 (*i.e*., 4 rows 5 columns) 20 μL drops of ~ 2 × 10^3 ^CFU mL^-1 ^of diluted *S. aureus *(grown in BHI broth overnight at 37°C) to each plate, making sure that a pipette tip mark indicated the center of each drop to locate where to dispense the *Np *solution. After drying, 20 μL of each *Np *concentration (up to *ca*. 800 nM) was added on top of each air-dried, organism-loaded drop. Growth at 37°C was checked daily for at least a week. Each such experimental procedure was replicated thrice using a fresh culture.

### 96-well microtitre plate growth assay procedures

Dilutions using liquid growth media (BHI) as the diluent were made from refrigerated (at least one day and up to 2 weeks), stationary-phase *Staphylococcus aureus *(Gram-positive coccus), *Salmonella *Typhimurium (Gram-negative bacillus), or *Escherichia coli *O157:H7 (Gram-negative bacillus) cultures grown in BHI. The sterile BHI broth was either fresh (< 1 month in the dark at room temperature) or the same medium which had been stored > 1 month. All media came from the same lot of starting material. Three hundred μL of each treatment combination ([*Np*] level and/or bacteria *C*_I_) were added to each well. Each specific bacterial concentration used is provided in Table or Figure legends. All freeze-dried keratin-capped *Np *levels were created by diluting with BHI. In order to avoid water condensation which might interfere with absorbance readings, the interior surface of microplate covers were rinsed with a solution of 0.05% Triton X-100 in 20% ethanol and dried in a microbiological hood under UV light [24]. All calculations took into account the small dilution upon adding the various *Np *solutions. A Perkin-Elmer HTS 7000+ 96-well plate reader was used for optical density (OD) measurements over time using: λ = 590 nm; temp = 37°C; time between points was either 10, 12 or 14 min and 110 data points were always collected.

After completion of any OD with time growth experiment, a tab-delimited text file was generated and data pasted into a Microsoft Excel spreadsheet formatted to display the data arrays as individual well ODs at each time point (OD[t]). OD growth curves were then curve-fitted to **Eq. 1 **which is a well-known sigmoidal function used in various physiological studies [23,26].

(1) $$OD_{590}=OD_{\text{F}}+\frac{OD_{\text{I}}-OD_{\text{F}}}{1+Exp\left[\left(t-t_{\text{m}}\right)k\right]}$$

**Figure 5:**
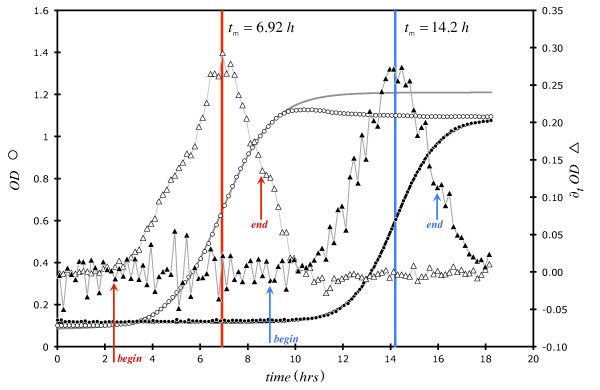
**Plot of optical density at 590 nm (circles) and associated first derivative (∂_t_OD, triangles) data associated with *S. aureus *growth (*C*_I _~ 10^4 ^CFU mL^-1^) at 37°C in BHI broth**. Open triangles/circles = negative control (beginning/ending arrows in red); closed triangles/circles ~10^-6 ^M freeze-dried keratin *Np*s (beginning/ending arrows in blue); starting bacteria concentration ~10^4 ^CFU mL^-1^.

In **Eq. 1**, OD_I _is the estimated initial optical density (0.05-0.1), OD_F _is the calculated final OD (0.8-1.2), *k *is a first-order rate constant (doubling time = τ = *Ln*[2] ÷ *k*), and *t*_m _is the time to OD = OD_F _÷ 2. The parameter *t*_m _is also the time where the maximum in the first derivative of OD[t] with time (∂_t_OD[t]) occurs and indicates the center of symmetry of the fitted **Eq. 1**. Typical OD[t] growth curves (*S. aureus*) are presented in Figure 5 which have been curve-fitted with **Eq. 1**. In this Figure, two growth curves (OD[t]: open circles = negative control; closed circles ~10^-6 ^M freeze-dried keratin *Np*s; *C*_I _= starting bacteria concentration ~10^4 ^CFU mL^-1^) are shown in time sequence along with ∂_t_OD[t] (triangle symbols). Notice that the calculated (from **Eq. 1**) *t_m_*s are approximately equivalent to the maxima in the ∂_t_OD[t] plots. In order to achieve the best fit we use only the OD[t] with time region which provides the most information (*i.e*., the exponential increase in OD[t]) and therefore have truncated all data and used only 5-10 points beyond the apparent *t*_m _to fit to **Eq. 1**. Such data abbreviation has been shown to have only minor effects on the growth parameters [23]. Figure 5 also shows the beginning and ending points of data truncation. All curve-fitting was performed using a Gauss-Newton algorithm on an Excel spreadsheet [27]. **Eq. 1 **appears to be generally useful with optically-based growth results since excellent fits were achieved when this equation was utilized to fit various [23,28] bacterial growth data.

We have recently [23] shown that (*E. coli*) doubling time (τ) values from OD[t] data fitted to **Eq. 1 **agreed with those obtained from manual plate counting with time. All values of *k *and *t*_m _reported herein are derived from such curve-fitting. Of course, *t*_m _can also be easily estimated from the x-axis value where the center of symmetry in ∂_t_OD[t] occurs.

During the log phase of growth [29], the rate of change in bacterial concentration with respect to time can be represented by the simple differential equation

(2) $$\frac{dC}{dt}=Ck;$$

in this relation, *k *is a first order rate constant, t is the growth time, and *C *is the bacterial concentration (CFU mL^-1^). Upon rearrangement, integration between initial (*C*_I _= *C*_0 _Φ _I_) and final (*C*_F _) values of *C *and solving for *C*_F _we see that

(3) $$C_{F}=C_{I}e^{\left(t-T\right)k};$$

where *T *is a time translation constant utilized to correct for the observed *lag *in cell growth (which is typically about 1 hour for our 3 bacterial species). In our usage, we assume that *C*_F _is the cell density at which the relationship between OD and *C *becomes non-linear, which is about 5 × 10^8 ^CFU mL^-1 ^for certain bacilli such as *E. coli *[23]. *C*_I _was measured by performing a drop plating procedure using 18-24 technical replicates per measurement (to minimize sampling error [30,31]) on the original stationary phase cultures which were diluted and dispensed into 96-well microtitre plates. The parameter *k *(an apparent first-order rate constant) was determined by curve fitting the OD[t] data to **Eq. 1**. Expressing **Eq. 3 **in terms of the time it takes to reach *C*_F _we see that

(4) $$t=k^{-1}Ln\left[\frac{C_{F}}{C_{I}}\right]+T.$$

We have chosen to express **Eq. 4 **in terms of *t*_m _which provides **Eq. 5 **(*i.e*., the value of t when *C *= *C*_F _÷ 2 and t = *t*_m_)

(5) $$t_{m}=k^{-1}Ln\left[\frac{C_{F}}{2C_{I}}\right]+T.$$

Knowing *t*_m_, *k*, *C*_I_, and *C*_F _we can estimate *T*. We calculate a corrected *T *(*T*_corr_) by merely assuming that the negative control in each set of *Np *experiments has a *T *= 1 hr. One common method [32] for determining *T *is by curve-fitting log-transformed plate count data with respect to time to another type of sigmoidal growth curve known as the Gompertz Equation (*e.g*., *Ln*[*C*] = α *Exp*[-*Exp*[*β *- *γt*]] +δ) where *T *is a function of both *β *and *γ *: *i.e*., *T *= [*β *- 1] γ^-1^± a propagated error term [32,33]. This kinetic method is very time consuming and proves difficult to observe a large number of treatments due to the time involved in collecting samples, plating, *etc*. However, using this manual technique we have found that both *E. coli *O157:H7 and *Salmonella *Typhimurium show similar lag times (*T *~ 1-1.5 hr) to *S. aureus *(*T *= 1.4 ± 0.49 hr) but somewhat larger *k *(*i.e*., a shorter τ). Since the apparent effect of both keratin- and citrate-capped *Np*s on *t*_m _could also result from a change in *C*_I _(*i.e*., cell death) we have estimated the probability (*P*_+_) for any growth occurring in the 96-well plates, assuming only changes in *C*_I _(*e.g*., with a *T *fixed at 1 hr)

(6) $$P_{+}=1-Exp\left[-C_{\text{I,calc}}V\right]$$

and

(7) $$C_{\text{I,calc}}=\frac{C_{\text{F}}Exp\left[k\left(1-t_{\text{m}}\right)\right]}{2}.$$

*C*_F _is iteratively evaluated in order to make *C*_I, obs _(negative control; based on enumeration of *C*_0 _using the 6 × 6 drop plate method [34]) = *C*_I, calc _inasmuch as *C*_I, obs_, *k *and *t*_m _have all been calculated empirically. **Eq. 6 **is a well-known expression for calculating the probability of observing positive growth (turbidity). When multiplied by *n*, the number of growth observations, **Eq. 6 **can be used to calculate the number of positives out of *n *observations of growth in most probable number (MPN) determinations. Briefly, **Eq**. **6 **can be produced when the partial first derivative of the *binomial probability distribution *function (*P*[*C*_I_]) with respect to *C*_I _is normalized to itself (*i.e*., $\partial_{C_{\text{I}}}P\left[C_{\text{I}}\right]÷P\left[C_{\text{I}}\right]$), set to zero, and the number of positive growth responses (*p*) calculated. When both sides of this relationship are divided by *n*, **Eq. 6 **is produced. **Eq. 7 **is just a rearrangement of **Eq. 5 **solving for *C*_I _and assuming *T *= 1 hr. For *S. aureus C*_F _is typically ca. 10^8 ^CFU mL^-1 ^and for the Gram-negative organisms, *C*_F _~ 5 × 10^8 ^[23]. Thus, in essence, *P*_+ _is the probability that the observed changes in *t*_m _could be due to perturbations in the *C*_I _in the presence of the *Np*s.

### Statistical Tests of Significance

In this work a "randomized complete block" [35] (also known as the "randomized block" [36]) design was used and replication was based upon either position on a plate (Table 1) or culture (all other reported experiments: *i.e*., a major source of variation in microbiology is the starting culture). We also used the well-known Tukey or *q*-value-based multiple range test [35 p 444, 36 appendix p 64]. For this multiple range test the *q*_0.05 _is multiplied by $\sqrt{EMS÷;b}=s_{\overset{¯;}{x}}$ (*EMS *= error mean square; *b *= number of blocks or true replicates encompassed by each treatment mean $\bar{x}$). The *q*-test is more stringent than many multiple range tests such as the Student's *t*. All figures presenting growth parameter data are provided with the observed average t_m _or *T*_corr _(average of 3 experiments) displayed as $\pm \;q_{0.05}s_{\bar{x}}÷2.$

## Competing interests

The authors declare that they have no competing interests.

## Authors' contributions

PI designed all of the experiments (with input from CC and JM), performed all calculations and statistical analyses, participated in running most of the experiments and drafted the manuscript. JM performed all the *Np *syntheses and characterizations and took part performing the various bioassays as well as helping to draft the manuscript. LN carried out all the *C*_I _enumeration assays and performed many of the OD growth experiments and helped draft the manuscript. YH, AG, and CC assisted in certain aspects of the experiments as well as drafting the manuscript. All authors read and approved the final manuscript.
